# Bottle Gourd Juice: Poison or Panacea

**DOI:** 10.7759/cureus.40886

**Published:** 2023-06-24

**Authors:** Sabir Hussain, Vivek Saini, Vaibhav K Varshney, Narendra Bhargava

**Affiliations:** 1 Gastroenterology, Dr. Sampurnanand Medical College, Jodhpur, IND; 2 Surgical Gastroenterology, All India Institute of Medical Sciences, Jodhpur, Jodhpur, IND

**Keywords:** bottle gourd juice, arid, emergency gastroenterology and endoscopy, systemic toxicity, poison

## Abstract

Ingestion of toxic bottle gourd juice, particularly the bitter one, may pose a significant risk to life if not treated in time. Notwithstanding its usefulness, people drink it routinely without concern for fruit quality, extraction hygiene, and mixture with other fruits. We report two cases of bottle gourd juice poisoning with severe abdominal pain and hematemesis. On evaluation, patients were hypotensive with associated esophagitis, pangastritis, and duodenitis. After conservative management, both were discharged after five days of hospitalisation. We conclude that the chances of bottle gourd juice poisoning are higher in water-stressed arid regions; hence, care should be taken on quality and quantity while consuming it.

## Introduction

Consumption of bottle gourd, aka Lauki/Ghiya (Lagenaria siceraria, Cucurbitaceae) juice, is rising in India. Older people, including millennials, are captivated by the growing obsession with such a healthy drink. Such juices are considered an elixir for weight loss, diabetes, and hypertension and to keep the liver, heart, and mental dexterity to their optimum [1,2]. Attractive and lucrative recipes, summaries, and benefits of bottle gourd from unverified sources are readily available over-the-counter and online, which seduce people to drink them as cheap and harmless products.

Bottle gourd is frequently used in Ayurveda and other folk medicines for its cardio-protective, diuretic, purgative, aphrodisiac, antidote to certain poisons, and cooling effects [1]. Though no scientific study exists, some people also believe the juice to have antihyperlipidemic, analgesic and anti-inflammatory, diuretic, and cardioprotective properties [2-4]. Despite such benefits and uses, bottle gourd juice may cause untoward symptoms and becomes dangerous to life because there is no scientific guidance on the quantity and quality of juice consumed. Several studies have been published on bottle gourd poisoning, mostly from India and a few from outside. Three deaths were also reported, therefore, considering the prospective seriousness of this hazard, an Indian Council of Medical Research (ICMR) expert committee was constituted to study and give recommendations [5]. Here, we discuss two cases of bottle gourd juice poisoning managed conservatively from western India.

## Case presentation

Case 1

A 38-year-old male presented in the Department of Gastroenterology at Dr. Sampurnanad Medical College, Jodhpur, with complaints of three episodes of hematemesis (~50 mL) with pain in the upper abdomen. Symptoms started one hour after consuming 100 mL of bitter bottle gourd juice in the morning. A thorough history was taken to rule out a variceal, peptic ulcer, binge alcohol intake, and corrosive ingestion-induced hematemesis. His consciousness level on Glasgow Coma Scale (GCS) was 15/15, pulse rate (PR) was 124 beats/min, respiratory rate was 32/min, blood pressure (BP) was 80/50 mmHg, oxygen saturation (SpO_2_) was 97%, and afebrile. On general examination, he had signs of dehydration evident by a sunken eyeball, dry tongue, and reduced urine output. Systemic examination was unremarkable. A comprehensive effort was put in to stabilise him and formulate a diagnosis. He was monitored in the intensive care unit with the initiation of intravenous (IV) fluids, a prophylactic broad-spectrum antibiotic, proton-pump inhibitors (PPI) infusion, anti-emetics, an opioid analgesic, inotropic support, catheterisation, blood sampling, electrocardiography (ECG), and vitals monitoring was done post-haste.

Initial laboratory investigations showed signs of severe dehydration: Haemoglobin - 20.7 g/dL, total leucocyte count (TLC) - 32,290/mm^3^, red blood cells (RBCs) - 6.66/uL, hematocrit (HCT) - 60.6%. Liver function test showed total bilirubin - 2.56 mg/dL, aspartate aminotransferase (AST) 1449 U/L, and alanine transaminase (ALT) - 859 U/L. Serum creatinine was slightly raised (1.46 mg/dL), with blood urea being 64 mg/dL. Serum sodium, potassium, blood sugar, and coagulation profile were within normal limits. The chest radiograph with ultrasound of the abdomen was normal. ECG shows sinus tachycardia and Troponin I was within limits. Overnight, his vitals improved, and the pain subsided, although he had one episode of hematemesis.

**Figure 1:**
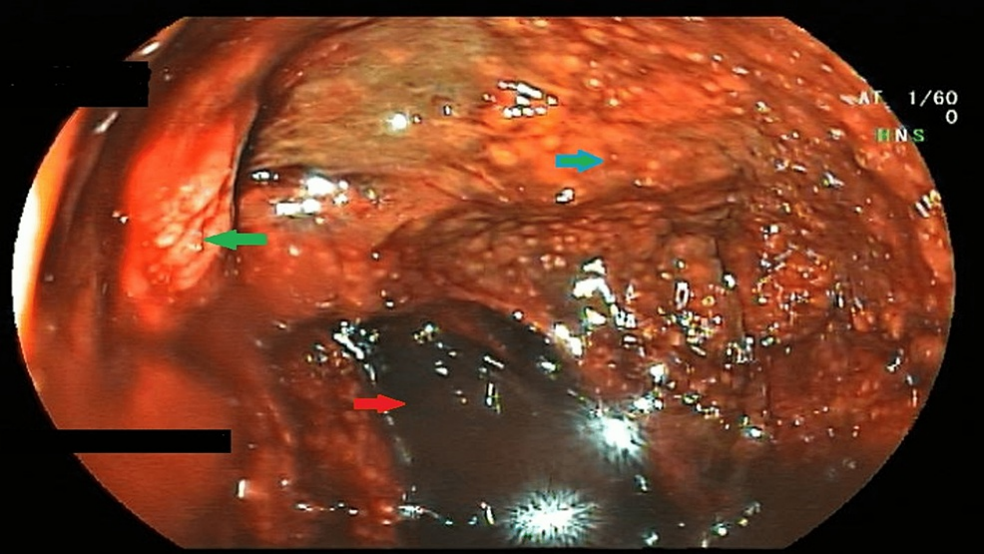
Endoscopic image of the stomach The image showing altered blood (red arrow) in the stomach with inflamed, edematous, erythematous gastric mucosa (blue arrow) with intervening ulcerations (green arrow).

On the second day of admission, he underwent esophagogastroduodenoscopy (EGD), which showed grade 2 esophagitis, pangastritis with altered blood in the stomach, and severe duodenitis (Figure 1).

On subsequent days, the patient was asymptomatic with stable vital parameters. He was kept on a soft diet orally. Repeated laboratory investigations showed a trend toward normalisation of haemoglobin, hematocrit, and liver enzymes as dehydration subsided. On the fifth day, the investigations showed significant improvement with AST/ALT - 98/110 U/L and TLC - 12300/mm^3^. The patient was discharged on oral PPI, antibiotics, anti-emetics, and opioid analgesics for five days. After a month, he was asymptomatic with normal routine investigations.

Case 2

A 58-year-old male known case of coronary artery disease and diabetes presented with complaints of recurrent episodes of hematemesis, pain abdomen, and 3-4 episodes of hematochezia since morning. Symptoms started 30 mins after consumption of bitter bottle gourd juice. At the time of presentation, his GCS was 13/15, BP 86/60 mmHg, PR 118/min, SpO_2_ 95%, with cold extremities, reduced skin turgor, and dry tongue. ECG, cardiac markers, blood sugars, and urine ketones ruled out any evidence of an acute coronary event and diabetic ketoacidosis. Computed tomography of the head was also unremarkable. Considering the history of consumption of bitter juice, the patient was investigated and managed accordingly. On investigation, his hemoglobin - 11.2 g/dL, TLC - 26,600/mm^3^, RBCs - 4.5/uL, and HCT - 36%. This is contrary to the hemoconcentration findings of Case 1 but could be because of hematemesis. Liver function test showed total bilirubin- 1.9 mg/dL, AST- 668 U/L, ALT - 750U/L. Renal function, serum electrolytes, and coagulation profile were within normal limits. EGD showed charring of the oesophagus, stomach, and duodenum mucosa, similar to Figure 1. Pantoprazole infusion, antiemetics, and IV fluids were given along with syrup sucralfate. He improved symptomatically with conservative management, and his lab investigations were normalised. He was discharged from the hospital after five days.

## Discussion

Jodhpur lies in the western part of Rajasthan state, an arid climate region of India characterised by low rainfall, high evaporation, and high temperature, suitable to stimulate high levels of cucurbitacin in the bottle gourd during summer. Over-ripened fruit in such stressful regions may acquire high cucurbitacin levels. Usually, the bitterness of juice is thought to be an indicator of toxicity due to the presence of a toxic compound tetracyclic triterpenoid called cucurbitacin [6]. The toxin inhibits the bindings of cortisol to glucocorticoid receptors in He La cell, while its sub-type D is known to increase capillary permeability, leading to hypotension in mice. When these toxins enter the blood, they can cause hepatitis, pancreatitis, cholecystitis, and, at times, organ dysfunction [7,8]. It also contains an allergic compound (aerpenebyonolic acid), a ribosome-inactivating protein (flavone-C glycosides), and choline. It is estimated that 50 mL of bitter bottle gourd juice can produce complications, while 300 mL can be fatal [5,8]. Hence, it is advisable to restrict the daily intake of juice to less than 30 mL.

Owing to its beneficial effects on heart diseases, liver diseases, hypertriglyceridemia, and diabetes, people drink it in varying amounts irrespective of taste, hygiene, and admixture [9]. But its unmindful consumption may lead to many health complications. Poisoning symptoms may start within a few minutes to 9 hours after consuming the bitter juice. Patients are reported with the main complaint of severe abdominal pain, vomiting, hematemesis, and diarrhoea. They might also be in shock, altered sensorium, or hypertension, which sometimes becomes serious about calling for surgery and even leading to death. Hence, the ICMR task force has recommended that the consumption of bitter-tasting bottle gourd juice should be avoided, and in case of having such symptoms or any feeling of uneasiness, the person should report immediately to a nearby hospital [5].

Endoscopic findings reveal esophagitis, gastritis, duodenitis, erosions, ulceration, charring, and necrosis of the oesophagus, stomach, and duodenum. At times the patient may need a gastrectomy [10]. Biochemically, the patients show elevated liver enzymes, raised hematocrit, and leukocytosis as a sign of dehydration.

Table 1 depicts the list of patients reported to have toxicity due to the consumption of bitter bottle gourd.

**Table 1:** A list of publications that reported toxicity due to bitter bottle gourd consumption Hb - haemoglobin (g/dL), IV - intravenous; PPI - proton-pump inhibitor; TLC - total leucocyte counts (/mm^3^); PLT - platelets count (/mm^3^), RR - respiratory rate(/minute); BP - blood pressure; RBC - red blood cells (cells/uL); PCV - packed cell volume in %; AST - aspartate transaminase; ALT - alanine transaminase; BL - bilirubin in mg/dL, Cr - serum creatinine (mg/dL); NA - not available

S. No.	Authors (years)	No. of cases	Clinical symptoms and signs	Notable laboratory derangement	Distinctive endoscopic findings	Treatment (days of hospitalisation)
1	Lee WT et al. 2022 [11]	1	Vomiting, hematemesis, diarrhoea, and abdominal pain Tachycardia, Tachypnoea	Hb-13.9	Pangastritis and duodenitis	IV fluids, PPI, Antiemetics, and packed cell transfusion (5 days).
2	Chavda DM et al. 2021 [12]	1	Vomiting, hematemesis, altered sensorium. Tachycardia, Hypotension, Tachypnoea	NA	NA	IV fluids, antibiotics, and supportive treatment.
3	Keyur B et al. 2020 [10]	1	Abdominal pain, hematemesis, and melena Tachycardia, Hypotension	TLC-18000, PLT-185000,	Charring of mucosa upper oesophagus to pylorus, posterior gastric necrosis patch.	Gastrectomy with closure of duodenal stump and feeding jejunostomy (8 days).
4	Rathi PM et al. 2016 [13]	1	Vomiting, hematemesis, and abdominal pain	RR-18, BP-110/55mmHg, Hb-17.9, TLC-33200, RBC-7.3, PCV-53.3,	Pangastritis, severe duodenitis.	IV fluids, PPI, Antiemetics (5 days).
5	Verma A et al. 2015 [7]	1	Abdominal pain, vomiting some with fresh blood, dehydration.	Hb -8.8, TLC -22 010, RBC-7.32 million, PCV-58.3%, AST:611, and ALT: 613	Esophagitis, pangastritis and severe duodenitis.	IV fluids, antibiotics, antiemetics, PPIs (4 days).
6	Khatib KI et al. 2014 [14]	1	Profuse vomiting, bloody diarrhoea and altered sensorium Tachycardia; Hypotension, Tachypnoea Oliguria	Impaired renal and liver functions	Inferred gastrointestinal bleed	IV fluids, antibiotics, PPI, Hydrocortisone (5 days).
7	Ho CH et al. 2014 [6]	5 (one family)	Abdominal cramping, tenesmus, diarrhoea, hematemesis. Tachycardia, Hypotension	Hb 14.6, PLT-164000, Potassium-3.4 mEq/L	NA	IV fluids, crystalloids and potassium supplementation (for four patients 3 hours and for one patient after 2 days).
8	Puri R et al. 2011 [8]	15	Abdominal pain, vomiting, hematemesis, diarrhoea and upper gastrointestinal bleed.	Hb-9 to22; TLC-5200 to 19900, Cr-0.7 to 2.6; BL-0.9 to 2.8; PCV-52 to 62.	Hyperemia, erosions and ulcerations in oesophagus, stomach and duodenum	IV fluids, dopamine and/or noradrenaline, PPIs, and blood transfusion (1-5 days).
9	Hussain S et al. (Present report)	2	Hematemesis, abdominal pain, loose stool, oliguria. Tachypnoea, Tachycardia, Hypotension	Hb-20.7, TLC-32,290, AST-1449; ALT-859	Severe gastritis with duodenitis.	IV fluids, PPI infusion, antiemetics.

Upper gastrointestinal symptoms like vomiting, hematemesis, abdominal pain and diarrhoea are the most common symptoms of bottle gourd poisoning [6-8,10-14]. In some cases, tachycardia, hypotension, and tachypnoea are also noted. Symptoms are often complex and may deceive clinicians into pondering gastrointestinal sepsis or cardiac event. ICMR guidelines recommend that such patients should be admitted and general supportive care should be provided immediately to maintain the haemodynamics and electrolyte balance. A nasogastric tube should be placed for gastric lavage and to assess gastrointestinal bleeding. PPTs should be started, and treatment for other complications should be given [5]. Further, the attending clinician progressively eliminates the possible differentials to reach the final diagnosis of bottle gourd juice poisoning. Depending upon the degree of mucosal injury, haemoglobin may fluctuate between 9 and 22 [8]. There is no specific antidote; however, patients are managed conservatively through IV fluids, antibiotics, antiemetics, and hydrocortisone as per the requirement and conditions of the patients [6,8,13]. The hospital stay in uncomplicated cases varies between one and four days except when surgical intervention is required, which may prolong the stay by up to eight days.

## Conclusions

Awareness about the likely hazards of Lauki juice is a must for public consumers. People should restrain from consuming bitter and ripped bottle gourds. We apprehend that poisoning may be more extensive than reported, and a large number of mild cases may not be coming to hospitals, especially in the water-stressed arid climate of western Rajasthan. Physicians, too, must be aware of the broad spectrum of symptoms and complications of such poisoning to avoid delays in proper treatment.
